# Comparison of Metformin and Repaglinide Monotherapy in the Treatment of New-Onset Type 2 Diabetes Mellitus

**DOI:** 10.7759/cureus.13045

**Published:** 2021-01-31

**Authors:** Amna Younas, Junaid Riaz, Tamoor Chughtai, Hamza Maqsood, Shifa Younus, Muhammad Qasim, Muhammad Saim, Shaheryar Qazi, Muhammad Khaliq, Mahnoor Fatima

**Affiliations:** 1 Medicine, Russells Hall Hospital, Dudley, GBR; 2 Medicine, Nishtar Medical University, Multan, PAK; 3 Cardiology, Nishtar Medical University, Multan, PAK; 4 Radiology, Nishtar Medical University, Multan, PAK

**Keywords:** diabetes mellitus, metformin, repaglinide, hba1c, rbs, fbs, monotherapy

## Abstract

Objectives

We intend to investigate the feasibility of using repaglinide as initial therapy in patients with newly diagnosed type 2 diabetes mellitus naive to the oral anti-hyperglycemic agents by validating the effects of repaglinide on glycemic control (HbA1c) in comparison with metformin monotherapy.

Methodology

This parallel-controlled, randomized study was carried at the outpatient department of a tertiary care hospital. Two-hundred patients of both genders with newly diagnosed type 2 diabetes mellitus were included. After taking relevant history and physical examination, we drew venous blood samples of each patient and sent them to the institutional laboratory for analysis of fasting blood sugar (FBS) levels, HbA1c, and lipid profile. We divided the patients into two subgroups based on the lottery method. Group A was prescribed metformin, and group B was prescribed repaglinide, while the dosages were adjusted according to the blood sugar levels. All data were analyzed using SPSS Software 25.0 (SPSS Inc., Chicago, USA). We reported the data as means along with the standard error.

Results

All patients completed the study. There was a decline in fasting blood glucose levels after three months of therapy, both in the metformin (135 mg/dl ± 6 mg/dl versus 115 mg/dl ± 7 mg/dl, p < 0.01) and repaglinide groups (145 ± 6 mg/dl versus 122 ± 6 mg/dl, p < 0.01). Similarly, significant reductions in HbA1c were seen in both metformin (7.12 ± 0.15% versus 6.67 ± 0.06%, p < 0.01) and repaglinide treatment groups (7.83 ± 0.67% versus 6.81 ± 0.07%, p < 0.01). After three months of treatment, body mass index (BMI) was significantly decreased in the metformin group (26.87±1.1 kg/m^2^ versus 25.11 ± 0.44 kg/m^2^, p < 0.05). However, the patients in repaglinide group demonstrated a very slight decrease in BMI (27.11 ± 1.6 kg/m^2^ versus 26.47 ± 0.40 kg/m^2^). On follow-up, we found a significant decrease in triglyceride levels in both groups (p < 0.01 and p < 0.05. respectively). We also found that only the patients in metformin group showed some improvements in total cholesterol and low-density lipoprotein (LDL) levels (p < 0.05).

Conclusion

Our study concluded that both metformin and repaglinide have similar anti-hyperglycemic effects. Repaglinide can be prescribed as an alternative drug to metformin in patients with new-onset diabetes mellitus.

## Introduction

Diabetes mellitus is a polygenic syndrome characterized by persistent hyperglycemia associated with derangement in metabolism [1]. In the modern era, due to exponential economic growth and lifestyle changes, diabetes mellitus has now become a global epidemic. As of 2016, the prevalence of type 2 diabetes mellitus is 11.7% in Pakistan. Males are affected more, with a prevalence of 11.20% as compared to females, who have a prevalence of 9.19% [2]. Diabetes mellitus leads to various life-threatening complications increasing both morbidity and mortality. Glycemic disturbances are the major risk factors for cardiovascular disease [3]. This increase in cardiovascular diseases ultimately leads to increased deaths [4]. Various research studies have concluded that diabetic complications are directly related to the degree of dysglycemia [5,6].

Twelve classes of anti-hyperglycemic agents are available, along with several fixed-dose combinations of oral agents and fixed-ratio combinations of injectable agents, all with complementary mechanisms of action. According to the American Association of Clinical Endocrinology (AACE) Comprehensive Diabetes Management Algorithm, patients with new-onset type 2 diabetes mellitus (T2DM) should be on monotherapy following the lifestyle modification if the entry HbA1c level is <7.5 [7].

Among all kinds of anti-diabetic medications, metformin inhibits glucose uptake by the liver. It also increases peripheral glucose uptake and consumption. The UK Prospective Diabetes Study (UKPDS) revealed that obese patients with newly diagnosed type 2 diabetes taking metformin as monotherapy had good results. It not only reduced their HbA1c levels but also decreased the risk of diabetes-related endpoints significantly [8]. Due to its efficient blood-glucose-lowering ability, significant effects on body weight, and cardiovascular protectiveness [9], metformin is recommended as the first-line anti-hyperglycemic drug for type 2 diabetes mellitus [6].

Meglitinide is another anti-hyperglycemic class with benzoic acid in its structure [10]. Repaglinide is the most commonly used drug of this class. It decreases the blood glucose level by stimulating the release of insulin in a rapid-acting style. A previous study has shown that the repaglinide is efficiently involved in improving first-phase insulin secretion. It also regulates postprandial blood glucose levels [11]. Various studies have demonstrated that both metformin and repaglinide have equal efficacy in maintaining blood glucose levels and cardiovascular risk profile in patients with type 2 diabetes mellitus [12,13].

There are few studies comparing the effects of these two medications on glycemic control in patients with new-onset type 2 diabetes mellitus. We did this study to investigate the feasibility of using repaglinide as initial therapy in patients with newly diagnosed T2DM naive to oral anti-hyperglycemic agents by validating the effects of repaglinide on glycemic control (HbA1c) in comparison with metformin monotherapy.

## Materials and methods

Study setting

This study was carried out at the outpatient department of a tertiary care hospital in Multan.

Subjects, sample size, and sampling technique

Two-hundred patients of both genders with newly diagnosed type 2 diabetes mellitus were approached. Simple random sampling was done.

Study design

The research approach employed a parallel-controlled, randomized study to compare the efficacy of metformin and repaglinide in newly onset type 2 diabetes mellitus.

Inclusion criteria

Patients with newly diagnosed type 2 diabetes mellitus with age ranging from 25 to 60 years were included in this study.

Exclusion criteria

Those excluded from the study were patients having liver disease, kidney disease, ischemic heart disease, or any other acute or chronic disorder. Patients taking other hypoglycemic drugs, systemic or inhaled glucocorticoids, or any other medications known to interfere with glucose metabolism were also excluded.

Data collection procedure

After taking consent from the ethical committee of the hospital, this study was carried out in the outpatient department of the hospital. After taking relevant history and physical examination, we drew venous blood samples of each patient and sent them to the institutional laboratory for analysis of fasting blood sugar (FBS) levels, HbA1c, and lipid profile. We divided the patients into two subgroups based on the lottery method. Group A (n = 100, 78 males, 22 females, age = 49.3 ± 2.4 years, BMI = 26.87 ± 1.1 kg/m^2^) was prescribed metformin, and group B (n = 100, 65 males, 35 females, age = 53.1 ± 1.2 years, BMI = 27.11 ± 1.6 kg/m^2^) was prescribed repaglinide, while the dosages were adjusted according to the blood sugar levels. We designed a specialized proforma to handle all the study information. Patients were followed up after three months to record the glycemic index, and the values were recorded.

Data analysis

All data were analyzed using SPSS Software 25.0 (SPSS Inc., Chicago, USA). We reported the data as means along with the standard error. For comparison of values before and after three months of treatment, we employed independent t-tests. Paired t-tests were employed for comparison between groups. For consideration of results to be statistically significant, a p value of <0.05 was selected. Conclusions were made accordingly.

## Results

All patients completed the study. The demographic details and baseline investigation values of the patients of both groups are given in Table 1.

**Table 1:** Sociodemographic and clinical parameters of diabetic patients with new-onset type 2 diabetes mellitus.

Patients’ characteristics	Metformin group (n = 100)	Repaglinide group (n = 100)
Age, years (mean ± SD)	49.3 ± 2.4	53.1 ± 1.2
Gender (%)		
Male	78(78)	65(65)
Female	22(22)	35(35)
BMI, kg/m² (mean ± SD)	26.87 ± 1.1	27.11 ± 1.6
Diabetes duration, months (mean ± SD)	1.34 ± 2.1	1.1 ± 1.8
Fasting blood glucose concentration, mg/dl (mean ± SD)	135 ± 6	145 ± 6
HbA1C (%) (mean ± SD)	7.12 ± 0.15	7.83 ± 0.67

Fasting blood glucose concentration

**Figure 1:**
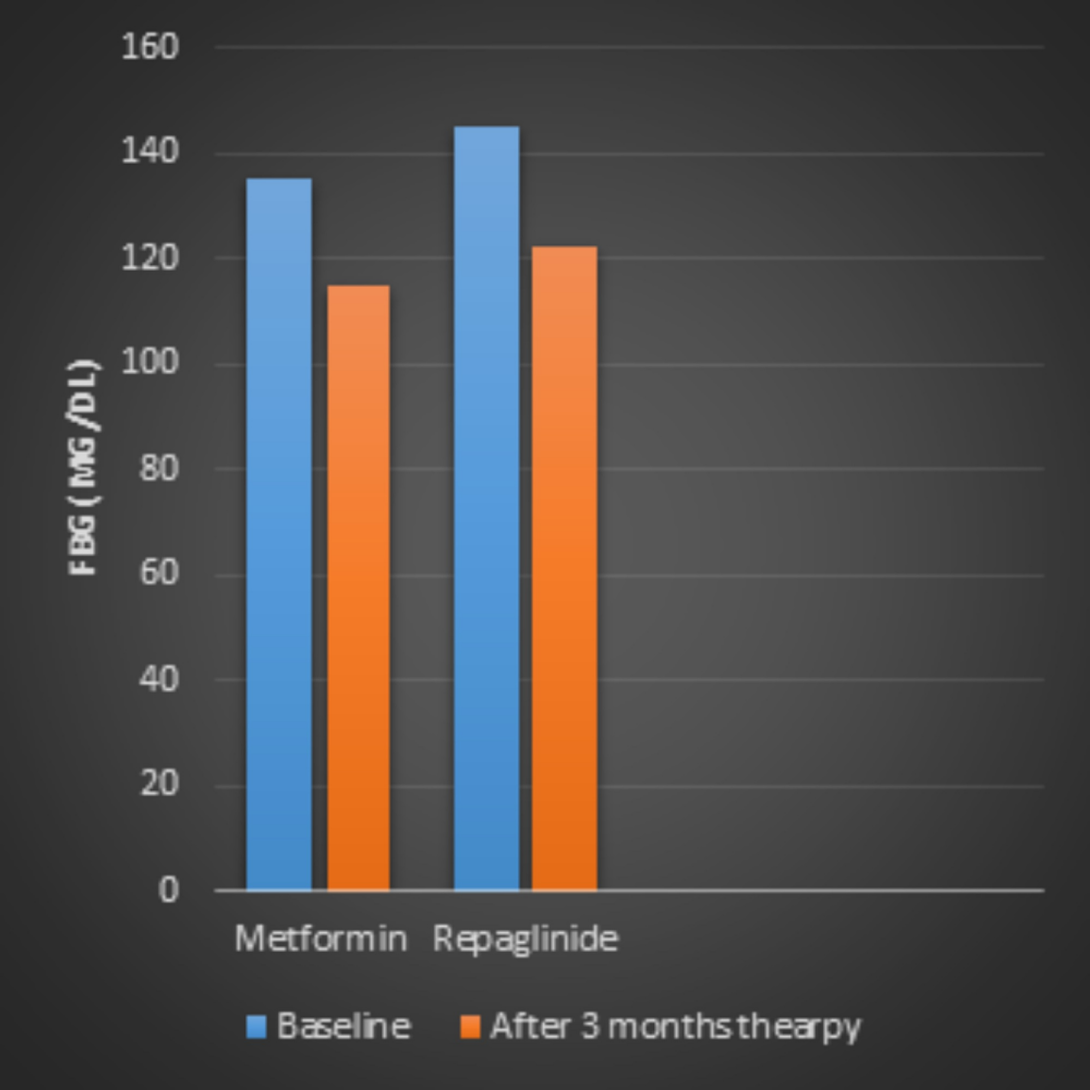
Fasting blood glucose levels in patients with type 2 diabetes before and after three-month treatment of metformin or repaglinide.

There was a decline in fasting blood glucose levels after three months of therapy, both in the metformin (135 mg/dl ± 6 mg/dl versus 115 mg/dl ± 7 mg/dl, p < 0.01) and repaglinide groups (145 ± 6 mg/dl versus 122 ± 6 mg/dl, p < 0.01). Fasting blood glucose did not show any statistically significant difference between the two groups (Figure 1).

HbA1c

**Figure 2:**
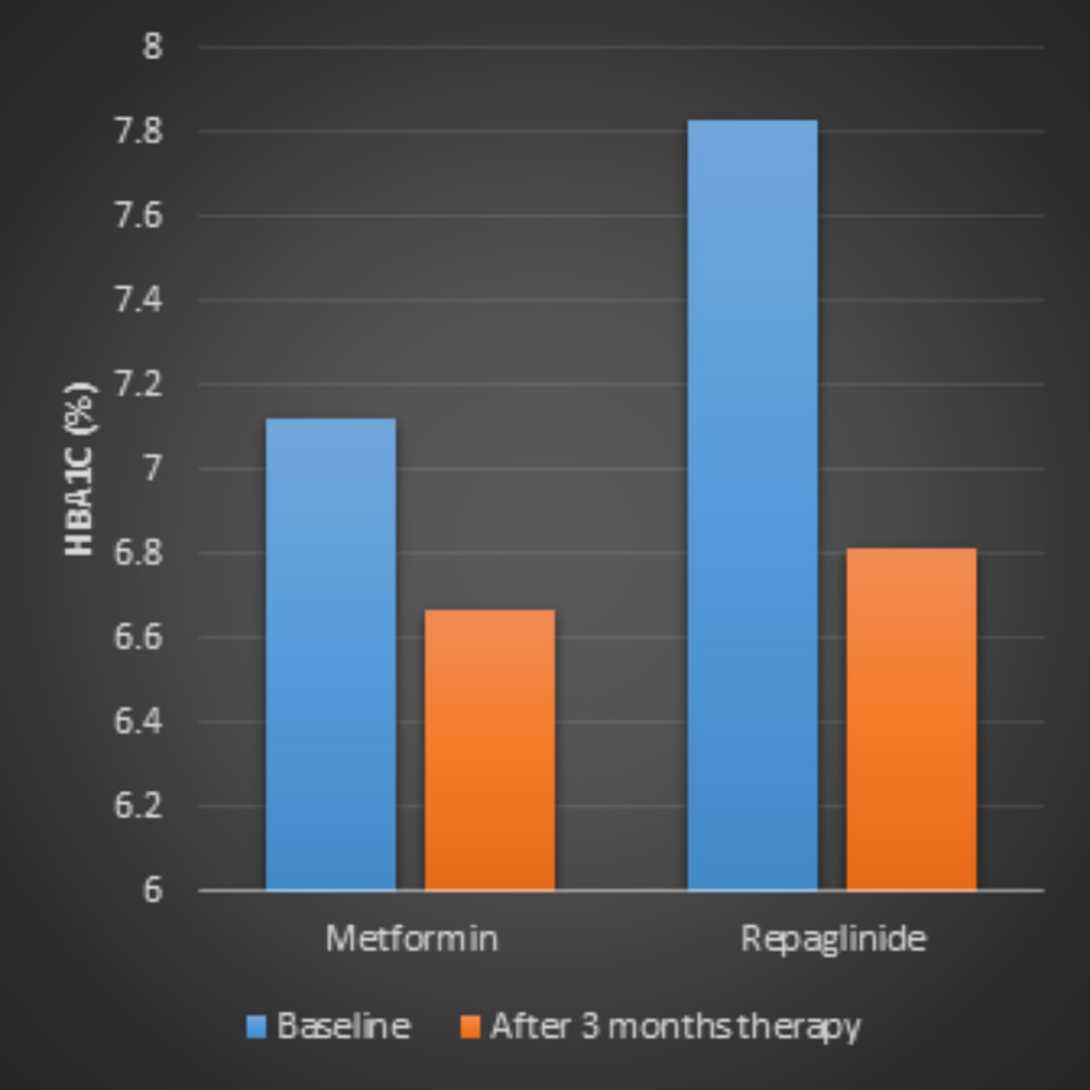
HbA1c levels in patients with type 2 diabetes before and after three-month treatment of metformin or repaglinide.

Significant reductions in HbA1c were seen in both metformin (7.12 ± 0.15% versus 6.67 ± 0.06%, p < 0.01) and repaglinide treatment groups (7.83 ± 0.67% versus 6.81 ± 0.07%, p < 0.01). The group taking repaglinide demonstrated higher reduction in HbA1c level (p < 0.01) (Figure 2).

Body mass index (BMI)

**Figure 3:**
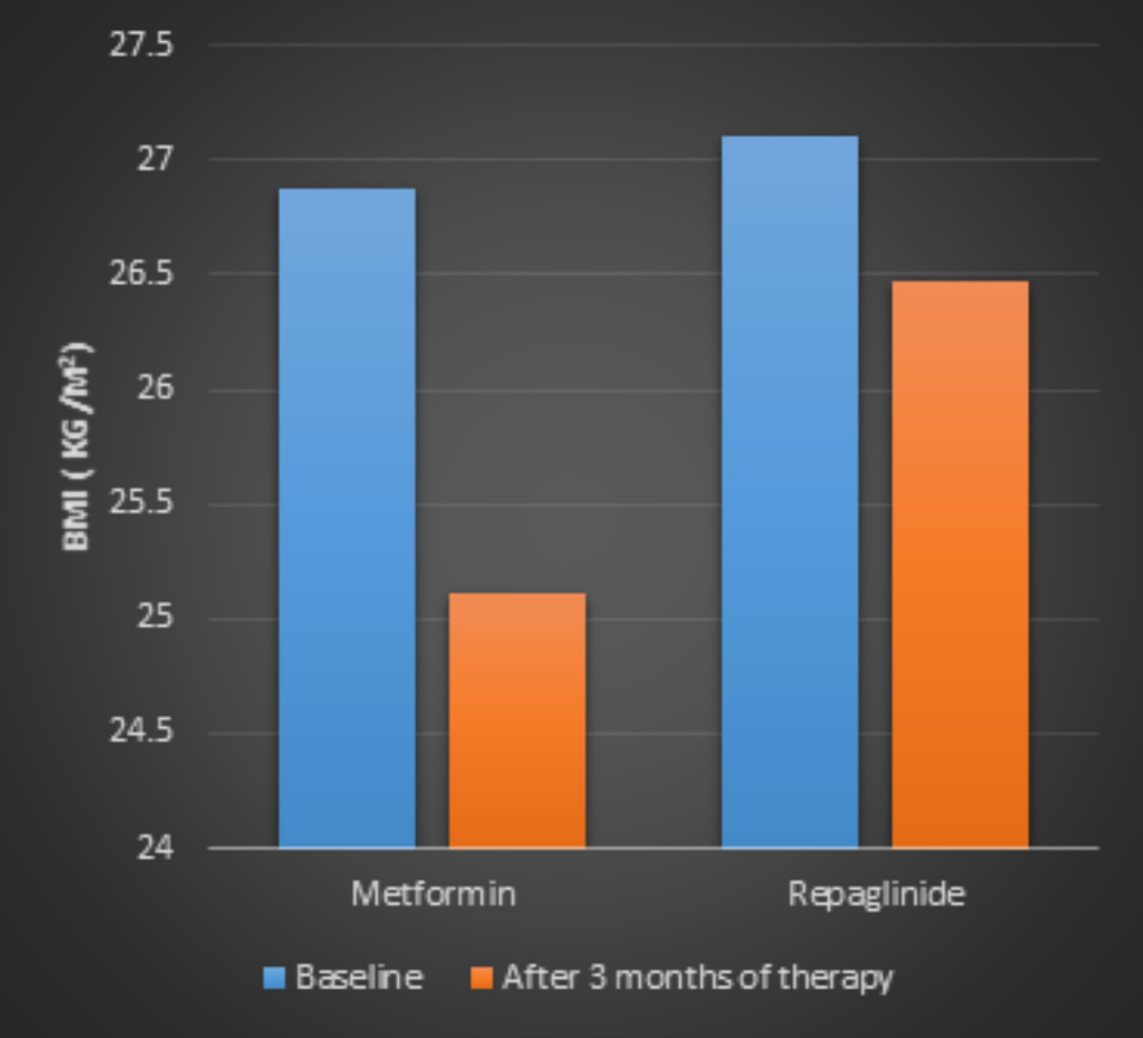
Body mass index (BMI) of patients with type 2 diabetes before and after three-month treatment of metformin or repaglinide.

After three months of treatment, BMI was significantly decreased in metformin group (26.87 ± 1.1 kg/m^2^ versus 25.11 ± 0.44 kg/m^2^; p < 0.05). However, the patients in the repaglinide group demonstrated a very slight decrease in BMI (27.11 ± 1.6 kg/m^2^ versus 26.47 ± 0.40 kg/m^2^). Repaglinide group did not show any statistically significant reduction of body mass index (Figure 3).

Serum lipids

On follow-up, we found a significant decrease in triglyceride levels in both groups (p < 0.01 and p < 0.05, respectively). We also found that only the patients in the metformin group showed some improvements in total cholesterol and LDL levels (p < 0.05). HDL-C was not affected by treatment with both drugs.

## Discussion

We showed that both metformin and repaglinide significantly decreased fasting blood glucose and HbA1c in newly diagnosed type 2 diabetes. The anti-diabetic effect of repaglinide was greater than metformin. There was a significant reduction in triglyceride levels in both groups, but only the metformin group showed improvement in total cholesterol and LDL levels.

The UKPDS has demonstrated that patients with good glycemic control have a lower risk of both micro- and macro-vascular complications of diabetes mellitus. HbA1c is the best laboratory investigation for the evaluation of glycemic control [8,14]. Both fasting and postprandial hyperglycemia contributes to different levels of HbA1c variations [15,16]. However, studies have shown that fasting blood glucose contributes fairly high to the overall glycemic control [5,16]. Furthermore, fasting hyperglycemia is more frequent than postprandial hyperglycemia in the prevalence of diabetes. Therefore, we compared the efficacy of metformin and repaglinide by evaluating the fasting blood glucose levels and HbA1c in patients with new-onset type 2 diabetes.

In the light of evidence-based medicine, almost all international guidelines recommend metformin as the only first-line oral anti-diabetic drug for patients with new-onset type 2 diabetes mellitus [6,17]. Due to contraindications and gastrointestinal complications occurring in more than 20% of patients taking metformin, other anti-hyperglycemic agents are considered first-line pharmacological therapy [18,19]. Our study showed that both metformin and repaglinide had a similar anti-hyperglycemic effect, which was in parallel with previous studies [5,12,13].

Hyperlipidemia is an isolated risk factor for a broad spectrum of cardiovascular diseases in patients with coexisting diabetes mellitus. The additive effect of oral anti-hyperglycemic agents on lowering lipid profile and body mass index is an extra benefit for patients with type 2 diabetes mellitus. Our study demonstrated that there was a significant reduction in triglyceride levels in both groups, but only the metformin group showed improvement in total cholesterol and LDL levels. These results are parallel with previous studies [5].

Our study has some limitations. First, the result might be biased by the open-label design in this study. Second, the mean BMI of patients in the metformin group was higher than those in the repaglinide group. Third, we did not measure plasma insulin concentrations and postprandial glucose levels as they can also be affected after three months of therapy. Finally, the duration of our study was short, along with a small study population.

## Conclusions

Our study concluded that both metformin and repaglinide have similar anti-hyperglycemic effects. Repaglinide can be prescribed as an alternative drug to metformin in patients with new-onset diabetes mellitus.

## References

[1] Maqsood H, Shakeel HA, Khan AR, Ali B, Shah SAY (2020). The descriptive study of anxiety levels among diabetics: insulin users versus non-insulin users. Int J Res Med Sci.

[2] Meo SA, Zia I, Bukhari IA, Arain SA (2020). Type 2 diabetes mellitus in Pakistan: current prevalence and future forecast. J Pak Med Assoc.

[3] Yang Z, Xing X, Xiao J (2013). Prevalence of cardiovascular disease and risk factors in the Chinese population with impaired glucose regulation: the 2007-2008 China National Diabetes and Metabolic Disorders Study. Exp Clin Endocrinol Diabetes.

[4] He J, Gu D, Wu X (2005). Major causes of death among men and women in China. N Engl J Med.

[5] Ma J, Liu LY, Wu PH, Liao Y, Tao T, Liu W (2014). Comparison of metformin and repaglinide monotherapy in the treatment of new onset type 2 diabetes mellitus in China. J Diabetes Res.

[6] Inzucchi SE, Bergenstal RM, Buse JB (2012). Management of hyperglycemia in type 2 diabetes: a patient-centered approach. Position statement of the American Diabetes Association (ADA) and the European Association for the Study of Diabetes (EASD). Diabetes Care.

[7] Tamez-Pérez HE, Proskauer-Peña SL, Hernŕndez-Coria MI, Garber AJ (2013). AACE comprehensive diabetes management algorithm 2013. Endocrine practice. Endocr Pract.

[8] UK Prospective Diabetes Study (UKPDS) Group (1998). Effect of intensive blood-glucose control with metformin on complications in overweight patients with type 2 diabetes (UKPDS 34). Lancet.

[9] Scarpello JH, Howlett HC (2008). Metformin therapy and clinical uses. Diab Vasc Dis Res.

[10] American Diabetes Association (2010). Diagnosis and classification of diabetes mellitus. Diabetes Care.

[11] Li Y, Xu L, Shen J (2010). Effects of short-term therapy with different insulin secretagogues on glucose metabolism, lipid parameters and oxidative stress in newly diagnosed type 2 diabetes mellitus. Diabetes Res Clin Pract.

[12] Derosa G, Mugellini A, Ciccarelli L, Crescenzi G, Fogari R (2003). Comparison of glycaemic control and cardiovascular risk profile in patients with type 2 diabetes during treatment with either repaglinide or metformin. Diabetes Res Clin Pract.

[13] Lund SS, Petersen M, Frandsen M (2008). Sustained postprandial decrease in plasma levels of LDL cholesterol in patients with type‐2 diabetes mellitus. Scand J Clin Lab Invest.

[14] Skyler JS, Bergenstal R, Bonow RO (2009). Implications of the ACCORD, ADVANCE, and VA diabetes trials: a position statement of the American Diabetes Association and a scientific statement of the American College of Cardiology Foundation and the American Heart Association. Diabetes Care.

[15] Monnier L, Lapinski H, Colette C (2003). Contributions of fasting and postprandial plasma glucose increments to the overall diurnal hyperglycemia of type 2 diabetic patients: variations with increasing levels of HbA1c. Diabetes Care.

[16] Riddle M, Umpierrez G, DiGenio A, Zhou R, Rosenstock J (2011). Contributions of basal and postprandial hyperglycemia over a wide range of A1C levels before and after treatment intensification in type 2 diabetes. Diabetes Care.

[17] American Diabetes Association (2013). Standards of medical care in diabetes—2014. Diabetes Care.

[18] Okayasu S, Kitaichi K, Hori A (2012). The evaluation of risk factors associated with adverse drug reactions by metformin in type 2 diabetes mellitus. Biol Pharm Bull.

[19] Ali S, Fonseca V (2012). Overview of metformin: special focus on metformin extended release. Expert Opin Pharmacother.

